# Sex‐Specific Differences in G_s_α‐Mediated Signaling Downstream of PTH1R Activation by Abaloparatide in Bone

**DOI:** 10.1002/jbm4.10695

**Published:** 2022-11-24

**Authors:** Srilatha Swami, Joshua Johnson, Lawrence A Vecchi, Matthew J Kim, Beate Lanske, Rachelle W Johnson, Joy Y Wu

**Affiliations:** ^1^ Division of Endocrinology, Department of Medicine Stanford University School of Medicine Stanford CA USA; ^2^ Department of Medicine, Division of Clinical Pharmacology Vanderbilt University Medical Center Nashville TN USA; ^3^ Vanderbilt Center for Bone Biology Vanderbilt University Medical Center Nashville TN USA; ^4^ Radius Health Boston MA USA

**Keywords:** ANABOLICS, GENETIC ANIMAL MODELS, OSTEOBLASTS, OSTEOPOROSIS, PTH/VIT D/FGF23

## Abstract

Teriparatide, recombinant parathyroid hormone (PTH[1‐34]), and abaloparatide, an analogue of PTH related‐peptide (PTHrP[1‐34]), are both anabolic medications for osteoporosis that target the PTH receptor PTH1R. PTH1R is a G protein–coupled receptor, and the stimulatory Gs protein is an important mediator of the anabolic actions of PTH1R activation in bone. We have published that mice lacking the α subunit of Gs in osteoprogenitors do not increase bone mass in response to PTH(1‐34). Unexpectedly, however, PTH(1‐34) still increases osteoblast numbers and bone formation rate in male mice, suggesting that PTH1R may have both Gs‐dependent and ‐independent actions in bone. Here we examine the role of Gs signaling in the anabolic actions of abaloparatide. We find that abaloparatide increases bone formation in male mice with postnatal deletion of G_s_α in Osx‐expressing osteoprogenitors (P‐G_s_α^OsxKO^ mice) but not in female P‐G_s_α^OsxKO^ mice. Therefore, abaloparatide has anabolic effects on bone in male but not female mice that appear to be independent of Gs‐mediated signaling. © 2022 The Authors. *JBMR Plus* published by Wiley Periodicals LLC on behalf of American Society for Bone and Mineral Research.

## Graphical abstract

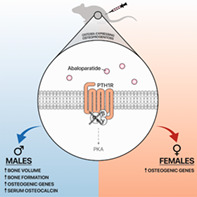

## Introduction

Osteoporosis is common and devastating. While the most frequently prescribed medications for osteoporosis are antiresorptives that inhibit osteoclast‐mediated bone resorption, anabolic therapies that target bone‐forming osteoblasts are crucial to any hope for a potential cure. Two FDA‐approved bone anabolic osteoporosis medications target the parathyroid hormone receptor (PTH1R): teriparatide, recombinant parathyroid hormone (PTH[1‐34]),^(^ ^1^ ^)^ and abaloparatide, an analogue of PTH related‐peptide (PTHrP[1‐34]).^(^ ^2^ ^)^

PTH1R is a G protein–coupled receptor that signals via a variety of G proteins. The stimulatory G protein Gs is an essential mediator of the anabolic effects of PTH1R activation in bone.^(^ ^3^, ^4^, ^5^ ^)^ Targeting of a mutant constitutively active PTH1R associated with Jansen metaphyseal chondrodysplasia to osteoblasts in mice leads to dramatic increases in trabecular bone volume^(^ ^3^ ^)^; this constitutively active PTH1R predominantly activates Gs‐dependent signaling.^(^ ^4^ ^)^

We have demonstrated that Gs signaling in osterix (Osx)‐expressing osteoprogenitors is required to increase trabecular bone induced by constitutively active PTH1R.^(^ ^5^ ^)^ Furthermore, mice lacking the α subunit of Gs in osteoprogenitors have severe osteoporosis and do not increase bone mass in response to anabolic (once‐daily) PTH(1‐34).^(^ ^5^, ^6^ ^)^ Unexpectedly, osteoblast numbers and bone formation rate increased in response to PTH in male mice,^(^ ^5^ ^)^ suggesting both Gs‐dependent and ‐independent actions of PTH1R in bone.

PTHrP is also a ligand for PTH1R, but PTH and PTHrP differ in PTH1R binding kinetics and downstream cyclic AMP (cAMP) activation. Although both PTH and PTHrP increase cAMP levels upon binding to PTH1R, PTH(1‐34) preferentially binds to a novel R^0^ (G protein–independent) PTH1R conformation, with continued cAMP accumulation even after internalization of the receptor‐ligand complex into endosomes.^(^ ^7^ ^)^ In contrast, PTHrP(1‐36) preferentially binds to the R^G^ (G protein–dependent) PTH1R conformation and activates cAMP only transiently at the cell surface.^(^ ^7^, ^8^ ^)^

Abaloparatide is a synthetic analogue of human PTHrP(1‐34) with even greater selectivity for the R^G^ PTH1R conformation and more transient cAMP accumulation.^(^ ^9^ ^)^ In a randomized, placebo‐ and active‐controlled trial in postmenopausal women with osteoporosis at high risk for fracture, abaloparatide increased total hip and femoral neck bone mineral density to a greater extent than teriparatide at 6, 12, and 18 months, with a lower incidence of hypercalcemia.^(^ ^2^ ^)^ The mechanisms by which these differences in PTH1R binding and activation translate into bone formation in vivo remain incompletely understood.

In this study, we sought to determine the contribution of G_s_α downstream of PTH1R in mediating the anabolic actions of abaloparatide in bone. We find that abaloparatide treatment increases bone formation in male but not female mice with postnatal deletion of G_s_α in Osx‐expressing osteoprogenitors (P‐G_s_α^OsxKO^ mice). Therefore, abaloparatide has sex‐specific anabolic effects on bone that can be mediated by Gs‐independent signaling in male mice.

## Materials and Methods

### Mice

The generation of Gsα^OsxKO^ mice lacking Gsα in Osx‐expressing osteoprogenitors has been described previously.^(^ ^6^ ^)^ Postnatal deletion of G_s_α (P‐G_s_α^OsxKO^ mice) was achieved by administering 100 μg/mL doxycycline in drinking water from conception until weaning. In both male and female P‐G_s_α^OsxKO^ mice, we find reduction of *Gnas* mRNA by approximately 50% in adult bone; because G_s_α is ubiquitously expressed, the remaining *Gnas* mRNA is likely due to expression in non‐osteoblast lineage cells.^(^ ^5^ ^)^ Groups of 10 adult male control and P‐G_s_α^OsxKO^ mice were treated with abaloparatide 40 mcg/kg/d 5 days per week (provided by Radius Health, Inc) or phosphate‐buffered saline (PBS) for 4 weeks starting at 10 to 13 weeks of age. Because of unexpected, impaired survival of female P‐G_s_α^OsxKO^ mice, groups of 6 adult female control and groups of 5 P‐G_s_α^OsxKO^ mice were treated with abaloparatide 40 mcg/kg/d 5 days per week or PBS for 4 weeks starting at 10 to 13 weeks of age. After 4 weeks, bone phenotypes were assessed by micro‐CT and histomorphometry. Because P‐G_s_α^OsxKO^ mice are of mixed genetic background, littermate G_s_α^fl/fl^ controls were used for all experiments except where otherwise specified.

### Micro‐computed tomography (micro‐CT) analysis

Femurs were scanned by a Scanco micro‐CT50 (SCANCO Medical AG, Brüttisellen, Switzerland) at 55 kVp and 145 μA intensity, 200 ms integration time, 1000 projections, with a 0.5 mm AI filter at a resolution of 10 μm/voxel. The trabecular region of interest (ROI) in the distal femoral metaphysis consisted of approximately 100 CT sections beginning 10% of total bone length distal to the growth plate and including the metaphysis. 3‐dimensional (3D) structural analyses were completed using the accompanying software to determine trabecular bone volume (BV/TV), trabecular number (Tb.N), trabecular thickness (Tb.Th), and trabecular spacing (Tb.Sp).

### Histomorphometry

Double calcein labeling was performed by injecting mice with 20 mg/kg calcein 3 and 10 days before euthanization. After femurs were scanned by μCT, they were dehydrated and embedded in methyl‐methacrylate and the distal femoral metaphysis was analyzed for dynamic bone formation rate (BFR), mineralizing surface (MS), and mineral apposition rate (MAR) using Bioquant (Nashville, TN, USA). Tibias from the same hindlimb were decalcified, processed, and embedded in paraffin. Histomorphometric analysis of decalcified/paraffin‐embedded tibias was performed using Bioquant to determine trabecular bone volume (BV/TV), trabecular number (Tb.N), trabecular thickness (Tb.Th), trabecular spacing (Tb.Sp), osteoblast surface (Ob.S), osteoblast number (N.Ob), osteoclast surface (Oc.S), and osteoclast number (N.Oc).

### Bone turnover markers

Serum was collected from each mouse for measurement of bone turnover markers. Fasting serum levels of osteocalcin (Quidel, San Diego, CA, USA) and TRAcP5b (Immunodiagnostic Systems Inc, Gaithersburg, MD, USA) were measured by enzyme‐linked immunoassay according to the manufacturers' protocols.

### Quantitative real‐time PCR

RNA was collected from flushed long bones of mice. Total RNA was isolated after homogenization using the Trizol reagent (Invitrogen, Waltham, MA, USA) according to the manufacturer's instructions. RNA (5 μg) was subjected to reverse transcription using the SuperScript III first‐strand synthesis kit (Invitrogen). Gene expression was determined by real‐time PCR using the CFX96 real‐time PCR detection system (Bio‐Rad Laboratories, Hercules, CA, USA) and the SYBR green qPCR kit (Bio‐Rad) using primers for *Runx2*,^(^ ^10^ ^)^ Osterix (*Sp7)*,^(^ ^10^ ^)^ collagen Iα1 (*ColIa1*),^(^ ^11^ ^)^ osteopontin (*Spp1*),^(^ ^10^ ^)^ osteocalcin (*Bglap*),^(^ ^12^ ^)^ sclerostin (*Sost*),^(^ ^13^ ^)^ bone sialoprotein (*Ibsp*),^(^ ^10^ ^)^ alkaline phosphatase (*Alpl*),^(^ ^10^ ^)^ matrix metalloproteinase 13 (*Mmp13*),^(^ ^14^ ^)^ osteoprotegerin (*Tnfrsf11b*),^(^ ^10^ ^)^ and RANKL (*Tnfsf11*)^(^ ^10^ ^)^ according to previously published protocols. Primer sequences are provided in Supplemental Table S1. Target gene expressions were normalized to β‐actin, and the relative changes in mRNA levels were assessed by the comparative CT method.

### Statistics

Statistical analyses were performed using GraphPad Prism 5 software (GraphPad Software, San Diego, CA, USA). Data were evaluated using 2‐way ANOVA with Tukey post hoc test for multiple comparisons. *P* values <0.05 were considered significant. Data were presented as box plots with median, interquartile range (25th to 75th percentile), bars representing range of data.

## Results

**Fig. 1:**
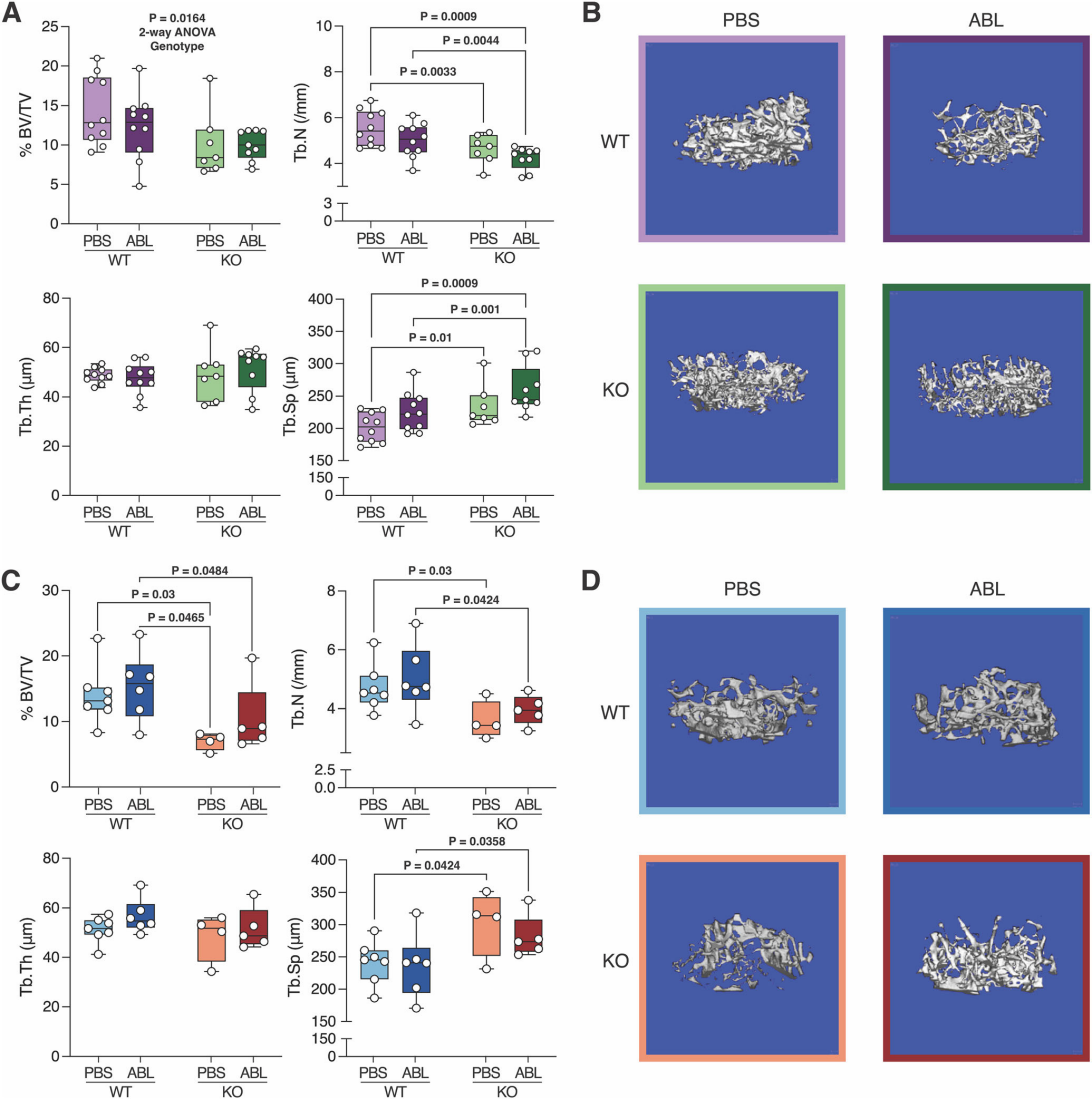
(*A*) Micro‐CT analysis of distal femur trabecular bone volume fraction (%BV/TV), trabecular number (Tb.N), trabecular thickness (Tb.Th), and trabecular spacing (Tb.Sp) of male mice (*n* = 10 for each group). (*B*) Representative 3D reconstruction of femur trabecular bone in male wild‐type (WT) and knockout (KO) mice treated with phosphate‐buffered saline (PBS) versus abaloparatide (ABL). (*C*) Micro‐CT analysis of distal femur %BV/TV, Tb.N, Tb.Th, and Tb.Sp of female mice (*n* = 6 for each group). (*D*) Representative 3D reconstruction of femur trabecular bone in female WT and KO mice treated with PBS versus ABL. Data in (*A*) and (*C*) are presented as box plots with median, interquartile range (25th to 75th percentile), and bars representing range of data.

Micro‐CT results from male mice are shown in Fig. 1*A*. There are no significant differences within the control or P‐G_s_α^OsxKO^ mice between saline and abaloparatide treatment. The 2‐way ANOVA is significant by genotype for bone volume fraction, and trabecular number is significantly decreased while trabecular spacing is increased in male P‐G_s_α^OsxKO^ mice relative to control mice, but there are no significant differences comparing saline versus abaloparatide. Representative 3D reconstructed micro‐CT images are shown for male mice in Fig. 1*B*.

Micro‐CT results for female mice are shown in Fig. 1*C*. Bone volume fraction and trabecular number are significantly decreased, while trabecular spacing is increased in female P‐G_s_α^OsxKO^ mice relative to control mice, but there are no significant differences comparing saline versus abaloparatide. Representative 3D reconstructed micro‐CT images are shown for female mice in Fig. 1*D*.

**Fig. 2:**
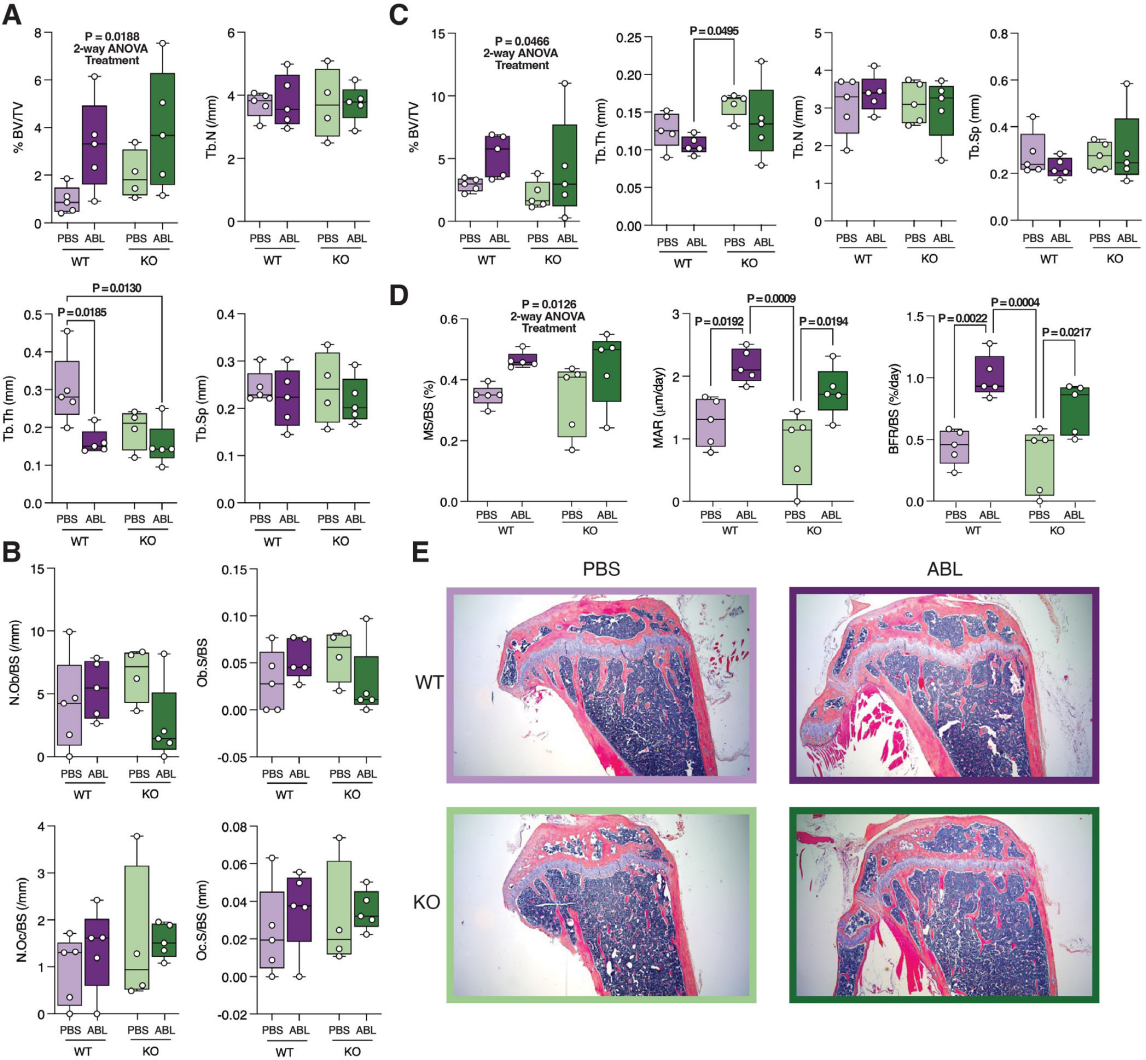
Histomorphometry analysis of male wild‐type (WT) and knockout (KO) mice treated with phosphate‐buffered saline (PBS) or abaloparatide (ABL). (*A*) Trabecular bone volume fraction (%BV/TV), trabecular number (Tb.N), trabecular thickness (Tb.Th), and trabecular spacing (Tb.Sp) and (*B*) osteoblast number (N.Ob/BS), osteoblast surface (Ob.S/BS), osteoclast number (N.Oc/BS), and osteoclast surface (Oc.S/BS) in decalcified/paraffin‐embedded tibias. (*C*) Trabecular %BV/TV, Tb.N, Tb.Th, and Tb.Sp in methyl methacrylate‐embedded distal femoral metaphysis. (*D*) Mineralizing surface (MS/BS), mineral apposition rate (MAR), and bone formation rate (BFR/BS) in methyl methacrylate‐embedded distal femoral metaphysis. *n* = 5 for each group of male mice. Data in (*A*–*D*) presented as box plots with median, interquartile range (25th to 75th percentile), and bars representing range of data. (*E*) Representative hematoxylin and eosin (H&E)‐stained sections from male WT and KO mice treated with PBS and ABL.

Histomorphometric analyses for male mice are shown in Fig. 2*A–D* and representative histology images in Fig. 2*E*. By histomorphometric analysis of undecalcified tibia, the 2‐way ANOVA is significant by treatment for BV/TV. Tb.Th is significantly reduced in control mice treated with abaloparatide (Fig. 2*A*). There are no significant differences in osteoblast number (N.Ob/BS) or surface (Ob.S/BS) or in osteoclast number (N.Oc/BS) or surface (Oc.S/BS) in male mice (Fig. 2*B*). By histomorphometric analysis of plastic‐embedded femora, the 2‐way ANOVA is significant by treatment for BV/TV and MS. Abaloparatide treatment significantly increases MAR and BFR in both control and P‐G_s_α^OsxKO^ male mice (Fig. 2*D*).

**Fig. 3:**
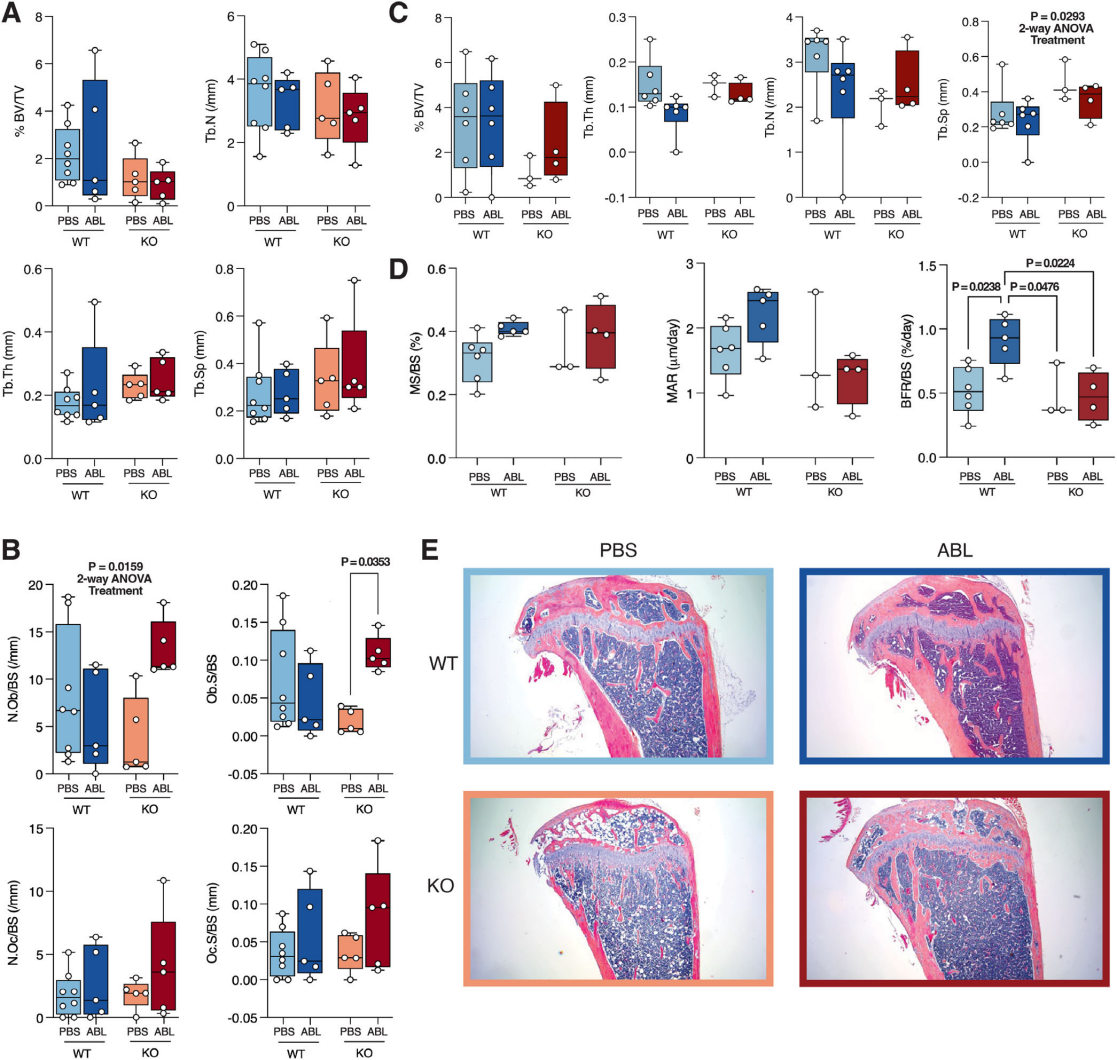
Histomorphometry analysis of female wild‐type (WT) and knockout (KO) mice treated with phosphate‐buffered saline (PBS) or abaloparatide (ABL). (*A*) Trabecular bone volume fraction (%BV/TV), trabecular number (Tb.N), trabecular thickness (Tb.Th), and trabecular spacing (Tb.Sp) and (*B*) osteoblast number (N.Ob/BS), osteoblast surface (Ob.S/BS), osteoclast number (N.Oc/BS), and osteoclast surface (Oc.S/BS) in decalcified/paraffin‐embedded tibias. *n* = 5 for each group of female mice. (*C*) Trabecular %BV/TV, Tb.N, Tb.Th, and Tb.Sp in methyl methacrylate‐embedded distal femoral metaphysis. *n* = 5 for each group of female mice, *n* = 3 for PBS‐treated KO mice. (*D*) Mineralizing surface (MS/BS), mineral apposition rate (MAR), and bone formation rate (BFR/BS) in methyl methacrylate‐embedded distal femoral metaphysis. Data in (*A*–*D*) presented as box plots with median, interquartile range (25th to 75th percentile), and bars representing range of data. (*E*) Representative hematoxylin and eosin (H&E)‐stained sections from female WT and KO mice treated with PBS and ABL.

Histomorphometric data for the females are shown in Fig. 3*A–D* and representative histology images in Fig. 3*E*. While variably apparent by histology, we have previously published that marrow adipocytes are increased in P‐G_s_α^OsxKO^ male and female mice.^(^ ^15^ ^)^ There are no differences in trabecular bone volume, trabecular number, thickness, or spacing in undecalcified tibias (Fig. 3*A*). The 2‐way ANOVA interaction is significant for osteoblast number and surface, which appears to be due to the dramatic increase in the P‐G_s_α^OsxKO^ mice after treatment with abaloparatide (Fig. 3*B*). In plastic‐embedded femora, the 2‐way ANOVA by genotype is significant for trabecular spacing (Fig. 3*C*). Abaloparatide increased BFR in control female mice only (Fig. 3*D*).

**Fig. 4:**
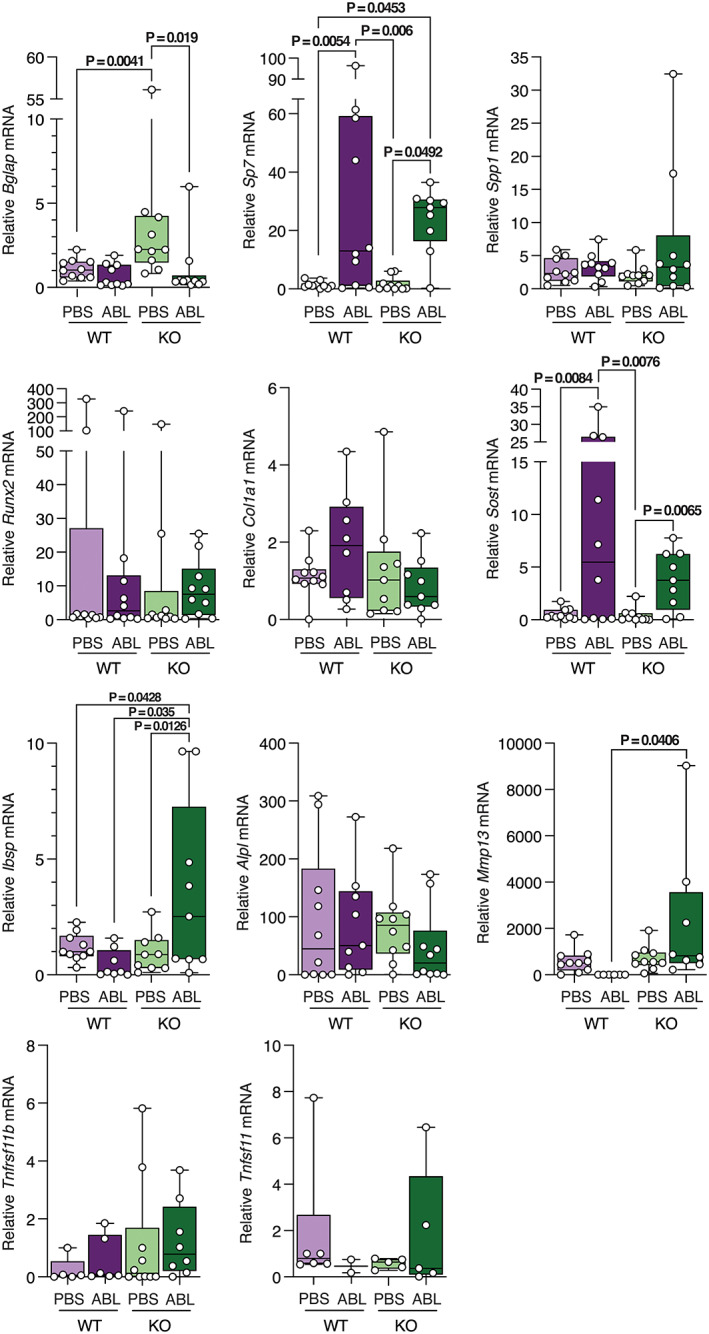
mRNA expression levels of *Bglap*, *Sp7*, *Spp1*, *Runx2*, *Col1a1*, *Sost*, *Ibsp*, *Alpl*, *Mmp13*, *Tnfrsf11b*, and *Tnfsf11* in bones of male wild‐type (WT) and knockout (KO) mice treated with phosphate‐buffered saline (PBS) or abaloparatide (ABL). Data presented as box plots with median, interquartile range (25th to 75th percentile), bars representing range of data.

**Fig. 5:**
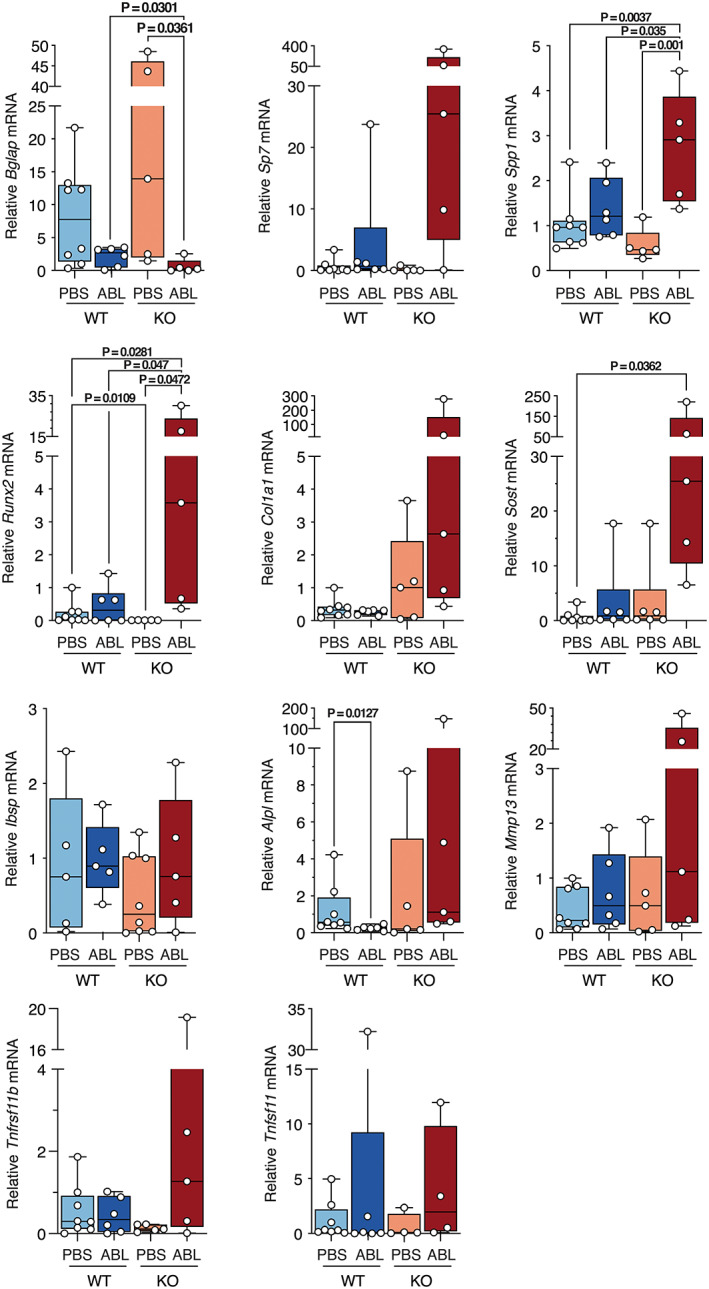
mRNA expression levels of *Bglap*, *Sp7*, *Spp1*, *Runx2*, *Col1a1*, *Sost*, *Ibsp*, *Alpl*, *Mmp13*, *Tnfrsf11b*, and *Tnfsf11* in bones of female wild‐type (WT) and knockout (KO) mice treated with phosphate‐buffered saline (PBS) or abaloparatide (ABL). Data presented as box plots with median, interquartile range (25th to 75th percentile), bars representing range of data.

We performed quantitative real‐time PCR (qPCR) on RNA isolated from bones of male (Fig. 4) and female (Fig. 5) mice to examine expression of osteoblast markers. In male mice, expression of *Bglap* is increased in KO mice treated with PBS but is not increased by abaloparatide. *Sp7* and *Sost* gene expression are increased by abaloparatide treatment in both control and P‐G_s_α^OsxKO^ male mice. Abaloparatide treatment increased expression of *Ibsp* and *Mmp13* only in P‐G_s_α^OsxKO^ male mice. No difference was noted in expression of *Spp1*, *Runx2*, *Col1a1*, *Alpl*, *Tnfrsf11b*, or *Tnfsf11* mRNA levels. In female mice (Fig. 5), abaloparatide decreased *Bglap* expression in P‐G_s_α^OsxKO^ females and *Alpl* expression in wild‐type (WT) females. Abaloparatide increased expression of *Spp1*, *Runx2*, and *Sost* in P‐G_s_α^OsxKO^ females.

**Fig. 6:**
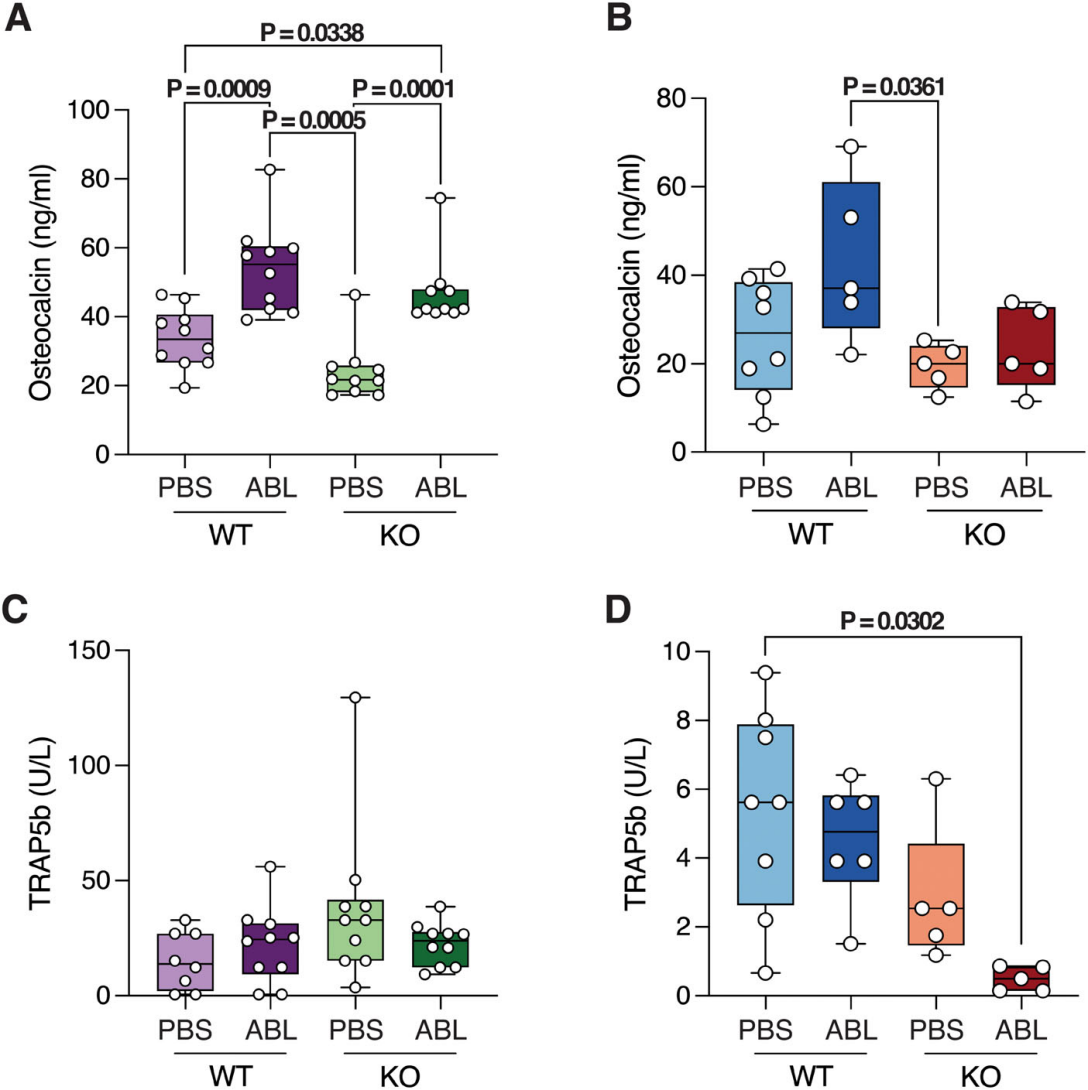
(*A, B*) Serum levels of osteocalcin from male and female wild‐type (WT) and knockout (KO) mice treated with phosphate‐buffered saline (PBS) or abaloparatide (ABL). *n* = 10 for each group of male mice, and *n* = 6 for each group of female mice. (*C, D*) Serum levels of TRAcP5b from male and female WT and KO mice treated with PBS and ABL. *n* = 10 for each group of male mice, and *n* = 6 for each group of female mice. Data presented as box plots with median, interquartile range (25th to 75th percentile), bars representing range of data.

Serum levels of osteocalcin, a marker of bone formation, were lower in both male and female P‐G_s_α^OsxKO^ mice at baseline (Fig. 6 *A*, *B*). Abaloparatide increased osteocalcin levels in both control and P‐G_s_α^OsxKO^ male mice but not in female mice. Serum levels of TRAcP5b, a marker of bone resorption, were decreased in abaloparatide‐treated female P‐G_s_α^OsxKO^ mice but otherwise did not differ between control and P‐G_s_α^OsxKO^ mice treated with abaloparatide (Fig. 6 *C*, *D*).

## Discussion

There are sex‐specific differences in the effects of abaloparatide on bone formation. Abaloparatide increases bone formation as reflected by BV/TV, MS, MAR, BFR, and serum osteocalcin in both control and P‐G_s_α^OsxKO^ males. Abaloparatide increases BFR in control but not P‐G_s_α^OsxKO^ females. Therefore, in male but not female mice, abaloparatide has anabolic effects on bone that appear to be independent of Gs‐mediated signaling.

In contrast to the histomorphometric data, micro‐CT analyses did not reveal notable differences. In the male mice, the increased bone volume detected by static histomorphometry but not micro‐CT could be due to differences in the anatomical region of interest used for analyses. The most pronounced increase in bone volume by histomorphometry was observed in the proximal tibial metaphysis, whereas micro‐CT was performed on femora. Histomorphometric analysis did also reveal a significant increase in bone volume in the femur with abaloparatide treatment, but this was less pronounced. It is also possible that if the bone was rapidly formed and not yet well mineralized, it was below the threshold for micro‐CT analysis.

We have previously reported that once‐daily PTH(1‐34) in P‐G_s_α^OsxKO^ mice fails to increase trabecular bone volume or cortical thickness in either male or female mice as measured by micro‐CT.^(^ ^5^ ^)^ However, histomorphometric analyses revealed that PTH increased osteoblast numbers and BFR in P‐G_s_α^OsxKO^ male mice only. We have also demonstrated that deletion of G_s_α in osteoprogenitors results in accelerated osteogenic differentiation.^(^ ^6^ ^)^ In calvarial osteoblasts harvested from G_s_α^fl/fl^ mice in which G_s_α was deleted in vitro by adenoviral Cre recombinase delivery, increased and accelerated mineralized nodule formation compared with control cells was further enhanced by PTH treatment, and PTH significantly increased expression of *Sp7*, *Col1a1*, and *Spp1*.^(^ ^5^ ^)^

Together these studies suggest that, at least in male mice, PTH1R activation may stimulate downstream signaling pathways independent of G_s_α. Other PTH1R‐coupled G proteins include Gq/G11 and G12/G13.^(^ ^16^, ^17^ ^)^ We have demonstrated that Gq/G11‐linked phospholipase C (PLC)‐protein kinase C (PKC) signaling is not required for the anabolic effects of PTH.^(^ ^5^ ^)^ In addition to PTH1R‐coupled G proteins, β‐arrestins have also been implicated in the anabolic actions of PTH on bone. β‐arrestins 1 and 2 are recruited upon PTH1R activation, facilitating receptor internalization and activation of ERK1/2 signaling.^(^ ^18^ ^)^ β‐arrestin2 is required for the full anabolic effect of PTH.^(^ ^19^ ^)^

The lower survival of female conditional knockout mice limits our ability to draw conclusions about the role of G_s_α‐signaling in mediating the anabolic actions of abaloparatide on bone in female mice, and our prior studies with PTH did not include histomorphometric analyses in female mice. Thus, additional studies are needed to determine whether activation of PTH1R by PTH has sex‐specific effects on osteoblast numbers and BFR.

We have previously published that marrow adipocytes are increased in P‐G_s_α^OsxKO^^(^ ^15^ ^)^ and PTH1R^Prx1KO^mice.^(^ ^20^ ^)^ PTH suppresses marrow adipocyte differentiation in favor of osteoblast differentiation.^(^ ^20^, ^21^ ^)^ Future studies will investigate the effects of abaloparatide on mesenchymal stem cell fate allocation.

## Disclosures

BL is an employee of Radius Health, Inc. All other authors state that they have no conflicts of interest.

## Author Contributions

**Srilatha Swami:** Formal analysis; investigation; writing – review and editing. **Joshua R Johnson:** Formal analysis; investigation; writing – review and editing. **Lawrence A. Vecchi:** Formal analysis; investigation; writing – review and editing. **Matthew Kim:** Formal analysis; visualization; writing – review and editing. **Beate Lanske:** Conceptualization; funding acquisition; project administration; resources; writing – review and editing. **Rachelle W Johnson:** Formal analysis; investigation; project administration; resources; writing – original draft; writing – review and editing. **Joy Y. Wu:** Conceptualization; formal analysis; funding acquisition; investigation; project administration; resources; writing – original draft; writing – review and editing.

## Supporting information


**Table S1.** Mouse Primer Sequences

## Data Availability

The genetically modified mice and data that support the findings of this study are readily available upon request.
