# Supplementary material for: Sex‐Specific Differences in Gsα‐Mediated Signaling Downstream of PTH1R Activation by Abaloparatide in Bone
Source: JBMR Plus. 2022 Nov 24;6(12):e10695. doi: 10.1002/jbm4.10695 (PMC9751656; doi:10.1002/jbm4.10695)
Supplement: Supplementary file 1 — Table S1. Mouse Primer Sequences [file JBM4-6-e10695-s001.docx]

**Supplemental Table 1**. Mouse primer sequences

| **Gene** | **Primer Sequence** | **Ref.** |
| --- | --- | --- |
| *Runx2* | Forward 5’ – AACCCACGGCCCTCCCTGAACTCT – 3’ | ^(10)^ |
|  | Reverse 5’ – ACTGGCGGGGTGTAGGTAAAGGTG – 3’ |  |
| Osterix (*Sp7*) | Forward 5’ – GGAGGTTTCACTCCATTCCA – 3’ | ^(10)^ |
|  | Reverse 5’ – TAGAAGGAGCAGGGGACAGA – 3’ |  |
| Collagen Iα1 (*Col1α1*) | Forward 5’ – CACCCTCAAGAGCCTGAGTC – 3’ | ^(11)^ |
|  | Reverse 5’ – GTTCGGGCTGATGTACCAGT – 3’ |  |
| Osteopontin (*Spp1*) | Forward 5’ – CTCCTTGCGCCACAGAATG – 3’ | ^(10)^ |
|  | Reverse 5’ – TGGGCAACAGGGATGACA – 3’ |  |
| Osteocalcin (*Bglap*) | Forward 5’ – TCTCTCTGCTCACTCTGCTGGCC – 3’ | ^(12)^ |
|  | Reverse 5’ – TTTGTCAGACTCAGGGCCGC – 3’ |  |
| Sclerostin (*Sost*) | Forward 5’ – CTTCAGGAATGATGCCACAGAGGT – 3’ | ^(13)^ |
|  | Reverse 5’ – ATCTTTGGCGTCATAGGGATGGTG – 3’ |  |
| Bone sialoprotein (*Ibsp*) | Forward 5’ – TACCGGCCACGCTACTTTCTTTAT – 3’ | ^(10)^ |
|  | Reverse 5’ – GACCGCCAGCTCGTTTTCATCC – 3’ |  |
| Alkaline phosphatase (*Alpl*) | Forward 5’ – CACGCGATGCAACACCACTCAGG – 3’ | ^(10)^ |
|  | Reverse 5’ – GCATGTCCCCGGGCTCAAAGA – 3’ |  |
| Matrix metalloproteinase (*Mmp13*) | Forward 5’ – TGATGAAACCTGGACAAGCA – 3’ | ^(14)^ |
|  | Reverse 5’ – TCCTCGGAGACTGGTAATGG – 3’ |  |
| Osteoprotegerin (*Tnfrsf11b*) | Forward 5’ – GGAACCCCAGAGCGAAACACAGT – 3’ | ^(10)^ |
|  | Reverse 5’ – CTTCTTCCCAGGCAGGCTCTCCAT – 3’ |  |
| RANKL *(Tnfsf11)* | Forward 5’ – GCTTCACTGCCCAGTCTCATCGTT – 3’ | ^(10)^ |
|  | Reverse 5’ – GGGCCGGTCCGTGTACTCATCCT – 3’ |  |
